# Effect of intralesional 5 fluorouracil injection in primary pterygium

**DOI:** 10.12669/pjms.321.8169

**Published:** 2016

**Authors:** Muhammad Saim Khan, Sidra Malik, Imran Basit

**Affiliations:** 1 Dr. Muhammad Saim Khan, MBBS, Armed Forces Institute of ophthalmology (AFIO), Rawalpindi, Pakistan; 2 Dr. Sidra Malik, MBBS, Armed Forces Institute of ophthalmology (AFIO), Rawalpindi, Pakistan; 3 Dr. Imran Basit, MCPS, FCPS, FRCS, Armed Forces Institute of ophthalmology (AFIO), Rawalpindi, Pakistan

**Keywords:** Pterygium, Recurrent pterygium, 5-Fluorouracil

## Abstract

**Objective:**

To determine mean change in visual acuity, corneal astigmatism and clinical appearance of pterygium after intralesional injection of 5-Fluorouracil.

**Methods:**

This was a Quasi experimental study conducted at Armed Forces Institute of Ophthalmology, Rawalpindi, Pakistan from June 2014 to May 2015. Total 68 eyes of 54 patients were included in the study. Patients were treated by injecting 0.1 ml of 5-FU (5mg) weekly injections for 04 weeks. All the patients underwent ophthalmic clinical examination that included Uncorrected distant visual acuity (UCVA), corrected distant visual acuity (CDVA), keratometery with Auto Ref-keratometer (RK-F1, Canon) and slit lamp examination before and 04 weeks after the last injection.

**Results:**

Total 68 eyes of 54 patients (18 females and 36 males) were treated with intralesional injection of 5 FU. Out of total, 30 were right eyes while 38 were left eyes. Age of patients ranged from 23 to 53 years with mean age of 39.2 ± 4.90 years. Mean UCVA and corneal astigmatism before treatment were 0.162 ± 0.167 and 2.12 ± 1.53 respectively while the same parameters 04 weeks after last injection of 5 FU were 0.166 ± 0.168 and 1.92±1.45 respectively. The magnitude of induced change in astigmatism was (0.235 ± 1.35). Ninety seven percent of the patients showed improvement in clinical appearance.

**Conclusion:**

Intralesional 5-FU injection results in significant clinical and cosmetic improvement of primary pterygium.

## INTRODUCTION

The word “Pterygium” is derived from the Greek word “pterygion” which means “wing” and this refers to its triangular shaped appearance in the interpalpebral conjunctiva. Pterygium is a superficial, elevated, fibrovascular growth of degenerative conjunctiva into the cornea and usually seen on nasal side.^1^ It affects males more commonly than females, however rarely affects individuals younger than twenty years of age.^2^,^3^ The etiology of pterygium is associated with variety of environmental and ocular risk factors like outdoor work, UV light exposure, occupational exposure to irritants, ocular inflammation and dryness. Countries located nearer to the equator have higher prevalence of pterygium due to higher exposure to UV light.^4^

The treatment for pterygium is indicated when there is progression threatening the visual axis, induced astigmatism, restriction of eye movement, chronic irritation or poor cosmesis, and the mostly the treatment is surgical excision. Excision of the pterygium with an autologous conjunctival graft is gold standard among the surgical techniques.^5^,^6^ Recurrence is the most undesirable treatment outcome of Pterygium surgery with overall recurrence rate of 18%.^7^,^8^ Various intraoperative and postoperative modalities including b-irradiation, topical thiotepa, mitomycin C and 5-fluorouracil (5-FU) have been introduced to decrease recurrence rate of pterygium surgery.^9^,^10^

5-FU is a pyrimidine analogue which interferes with DNA and RNA synthesis by inhibiting thymidylate synthetase enzyme and induces apoptosis in proliferating fibroblasts. It has been widely used in ophthalmology due to its antifibrotic and anti scarring properties.^10^-^12^

The rationale of conducting this study was to see the effect of 5-FU in primary pterygia without any surgical intervention. This may help in management of patients by reducing the symptoms and need for surgical excision.

## METHODS

This was a Quasi experimental study conducted at Armed Forces Institute of Ophthalmology, Rawalpindi, Pakistan from June 2014 to May 2015. We recruited 76 eyes of 60 patients by nonprobability convenience sampling but six patients were lost to follow up and finally we had 68 eyes of 54 patients. Included were the patients with age ranging from 20 to 60 years, who presented with watering, irritation, poor cosmetic appearance or decreased vision due to primary pterygium with an extent of 2 or more millimeter over cornea and thick, fleshy appearance in which episcleral vessels underlying the body of the pterygium were partly/totally obscured by fibrovascular tissue (Donald Grade 2 and 3). Patients with cataract, glaucoma, corneal scaring, trauma, or ocular surgery were excluded from the study.

Informed written consent was taken and all the patients underwent ophthalmic clinical examination that included uncorrected distant visual acuity (UCVA), corrected distant visual acuity (CDVA), keratometery with Auto Ref-keratometer (RK-F1, Canon) and slit lamp examination to grade the pterygium. Clinical appearance of pterygium was graded on the basis of Donald classification into three grades. Pterygia were labelled grade-1 if the underlying episcleral vessels were clearly visible, grade 3 if the vessels were completely obscured by the fibrovascular fleshy mass of pterygium and grade 2 if the vesseles were partly visible. All keratometery measurements were repeated three times and the mean was selected. Patients were recalled after 04 weeks of last injection and a detailed examination including UCVA, keratometery and slit lamp examination of pterygium were repeated.

### Procedure

We treated the patients by injecting 0.1ml of 5FU (5mg) weekly injections for 04 weeks. 5-FU solution is available in preformed strength of 50mg/ml in an ampule containing 10ml. The solution was injected into the belly of pterygium approaching from superior border toward center taking care not to puncture any large blood vessel. After injecting 0.1ml (5mg) of 5-FU, the needle was gently pulled back with least manipulation to avoid spillover. The injection was given under topical anesthesia (proparacaine 1%) in the outdoor patient department using a slit lamp. After the injection, topical 1–2 drops of chloramphenicol 0.5% thrice daily were advised for a week.

### Statisticalanalysis

Statistical package for social sciences (SPSS 22.0) for windows was used for statistical analysis. The continuous data was described in terms of mean ± SD (standard deviation). The induced change in astigmatism comparing the preoperative and postoperative measures were evaluated statistically with paired sample test (p ≤ 0.05 significance level). Preoperative and postoperative clinical appearance and grading of pterygium was analyzed by frequency distribution and Wilcoxon test was used to determine significance levels (P<0.05 significance level).

## RESULTS

**Fig.1:**
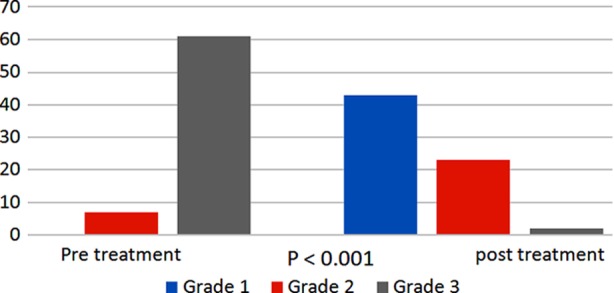
Frequencies of various grades of pterygium before and after treatment.

**Fig.2:**
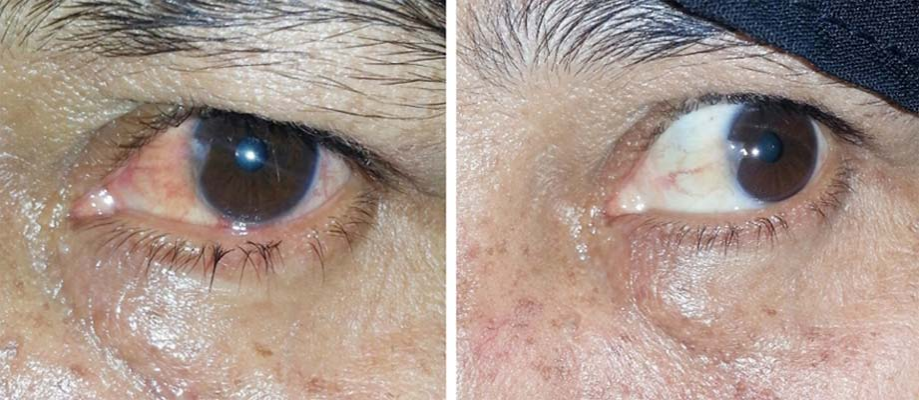
Showing clinical appearance of pterygium before and after treatment.

Total 68 eyes of 54 patients (18 females and 36 males) were treated with intralesional injection of 5 FU. Out of total, 30 were right eyes while 38 were left eyes. Age of patients ranged from 23 to 53 years with mean age of 39.2 ± 4.90 years (Table-I). UCVA of patients ranged from 0.00 to 0.47 with mean pretreatment visual acuity of 0.162 ± 0.167 and mean post-treatment visual acuity of 0.166 ± 0.168. Astigmatism as measured by Auto Ref-keratometer (RK-F1, Canon)before treatment was from 0.00 D to -7.50 with a mean of 2.12 ± 1.53 and same parameter, 04 weeks after last injection of 5-FU ranged from 0.00 DS to 6.00 D with a mean of 1.92±1.45. The magnitude of induced change in visual acuity and corneal astigmatism was 0.0039 ± 0.099 and 0.235 ± 1.35 respectively as shown in (Table-II). Patients were evaluated one month after the last injection and frequency of various clinical grades (Donald grading) was documented (Table-III). Comparison of pre and post clinical appearance is depicted in Fig.1 while Fig.2 presents pre and post treatment photographs of a patient.

**Table-I:** Mean values of various variables.

Variables	N	Min	Max	Mean±SD
Age	68	23	53	39.2±4.90
Pre treatment Visual acuity	68	0.00	0.47	0.16±0.166
Pre treatment Astigmatism	68	0.00	7.50	2.12±1.53
Post treatment Visual acuity	68	0.00	0.47	0.166±0.16
Post treatment Astigmatism	68	0.00	6.00	1.92±1.45

**Table-II:** Induced change in visualacuity and corneal astigmatism after treatment.

	Pre treatment	Post treatment	Induced change	P value
Visual acuity	0.16±0.166	0.166±0.16	0.0039±0.099	0.74
Corneal Astigmatism	2.12±1.53	1.92±1.45	0.235±1.35	0.15

**Table-III:** Frequency distribution of various grades of pterygium before and after treatment.

Grades	Pre treatment		Post treatment	

	N	%	N	%
Grade 1	0	0	43	63.2
Grade 2	7	10.2	23	33.8
Grade 3	61	89.8	2	2.9

## DISCUSSION

Pterygium is a common ocular surface disease which is characterized by abnormal epithelial and fibroblastic proliferation, invasion, matrix deposition and matrix remodeling.^13^ Clinically it progresses and migrates from periphery over the limbus towards center usually on nasal side leading to ocular irritation and decreased vision secondary to obscuration of visual axis and induced astigmatism.^13^,^14^ The exact mechanism is not known, however fibroblast proliferation is the prime mechanism of pterygium formation and surgery with or without adjuvant therapy is the mainstay of treatment.^14^ ‘Recurrence’ after surgical excision is a big concern so far in the management of pterygium and its incidence appears to increase with increasing number of operations.^15^ Fluorouracil (5-fluorouracil, 5-FU) was initially known for its use as anticancer drug due to its anti proliferative and anti fibroblastic mechanism. As the etiology of pterygium formation is fibroblastic proliferation, so it has been used as adjuvant for treatment of primary as well as recurrent pterygia.^16^ Various studies have depicted the role of intraoperative and postoperative intralesional administration of 5-FU to be safe and efficient in the treatment of pterygium and halting its recurrence.^17^,^18^ However, to the best of our knowledge no study so far has considered the role of 5-FU in primary pterygium without surgical intervention. We, in our study noticed clinically as well as statistically significant improvement in clinical appearance of pterygium. (Fig. 1 & 2). 97% of the patients had improvement in redness, congestion and fleshy appearance of the pterygium as evident from conversion to Donald’s grade 1 from grade 2/grade3. (Fig.1). Studies conducted by Razmjoo H et al.^19^ and Khan FA et al.^20^ concluded that surgical excision of pterygium results in improvement of visual acuity and induced corneal astigmatism, however, our results with 5-FU were inconsistent and the improvement in visual acuity as well as corneal astigmatism were not statistically significant as evident from Table-II.

Although use of 5-FU in filtering glaucoma surgery revealed adverse effects on corneal epithelial cells leading to keratopathy, we found 5-FU as being relatively safe for intralesional use especially the technique we followed for injecting the drug. Fortunately, only one of our patient developed punctate epithelial keratitis which resolved within a week after treatment with topical moxifloxacin.

Cosmesis is one of the most important concern and an important postoperative endpoint of pterygium patients for which they seek medical advice. Our results has shown that clinical appearance improves with intralesional 5-FU treatment. Moreover, those patients who are primarily concerned about the appearance of pterygium and those who are not willing for surgery can be good candidates for this treatment.

## CONCLUSION

The existing knowledge concerning the use of 5-FU in pterygium and its effect on clinical and cosmetic appearance encouraged us to conduct this study. We think our findings are important, however, further studies on similar large data sets are required to confirm the magnitude of changes we observed.
